# Bioactive Nanoparticles for Cancer Immunotherapy

**DOI:** 10.3390/ijms19123877

**Published:** 2018-12-04

**Authors:** Suchithra Poilil Surendran, Myeong Ju Moon, Rayoung Park, Yong Yeon Jeong

**Affiliations:** 1 Department of Biomedical Sciences, Biomolecular Theranostics (BiT) Lab, Chonnam National University Medical School, Hwasun 58128, Korea; 9pssuchithra@gmail.com; 2 Department of Radiology, Biomolecular Theranostics (BiT) Lab, Chonnam National University Medical School, Hwasun 58128, Korea; mjmoon2398@gmail.com (M.J.M.); parkry@daum.net (R.P.)

**Keywords:** cancer immunotherapy, bioactive nanoparticles, liposomes, PLGA nanoparticles, gold nanoparticles

## Abstract

Currently, immunotherapy is considered to be one of the effective treatment modalities for cancer. All the developments and discoveries in this field up to the recent Nobel Prize add to the interest for research into this vast area of study. Targeting tumor environment as well as the immune system is a suitable strategy to be applied for cancer treatment. Usage of nanoparticle systems for delivery of immunotherapeutic agents to the body being widely studied and found to be a promising area of research to be considered and investigated further. Nanoparticles for immunotherapy would be one of the effective treatment options for cancer therapy in the future due to their high specificity, efficacy, ability to diagnose, imaging, and therapeutic effect. Among the many nanoparticle systems, polylactic-co-glycolic acid (PLGA) nanoparticles, liposomes, micelles, gold nanoparticles, iron oxide, dendrimers, and artificial exosomes are widely used for immunotherapy of cancer. Moreover, the combination therapy found to be the more effective way of treating the tumor. Here, we review the current trends in nanoparticle therapy and efficiency of these nanosystems in delivering antigens, adjuvants, therapeutic drugs, and other immunotherapeutic agents. This review summarizes the currently available bioactive nanoparticle systems for cancer immunotherapy.

## 1. Introduction

The treatment for cancer has been studied and explored for decades. Advancements and breakthroughs in this area have changed the outlook of cancer treatment options. A number of therapeutic options are currently available for treatment, and many are under clinical trials. Cancer can be treated utilizing many methods, such as surgery, chemotherapy, radiotherapy, hormone therapy, and immunotherapy. Among these modalities, surgery has been used broadly as a treatment modality. However, the reoccurrence of tumors is the main drawback of this treatment option. The side effects of chemotherapy, hormone therapy, and radiotherapy are also considered to be a downside of the current cancer treatment. These kinds of drawbacks in treating the tumor have led to approaches for more specific targeting of the tumor site with fewer side effects [1,2].

Among the number of treatment options for cancer, immunotherapy is one of the effective methods due to its specificity in boosting our natural defense mechanism against cancer. The important milestone in the evolution of cancer immunotherapy is considered to be the manipulation of the immune system at the molecular and cellular levels [3,4,5]. After decades of efforts to determine the perfect immunotherapeutic option, vaccines, proteins such as antibodies and many drugs have been discovered. The innovation of biomaterials for the immune checkpoint blockade and CAR (chimeric antigen receptor) T cell response is considered to be a remarkable achievement in this field [6,7,8,9].

Both off-target side effects, cost, and lengthy processing are considered to be drawbacks of immunotherapy treatment methods [10,11]. The safety and efficacy of immunotherapy can be improved by incorporating different therapeutic agents into engineered biomaterials for targeting specific immune reactions. Currently used bioactive carriers are able to load or incorporate biological molecules and even cells. This approach could be utilized for the targeted delivery of antigens, vaccines, proteins, or other immunotherapeutic agents to the desired site. When compared to the conventional immunotherapy with antigens and adjuvants, the combination of conventional therapeutic strategies with current therapeutic strategies would be a suitable option. Conventional approach to treat cancer involved usage of antigens as well as adjuvants. The use of nanoparticles for delivery of these antigens, adjuvants, and other therapeutic agents resulted in the more specific targeting and better outcome in contrast to the conventional method [12,13,14].

The physicochemical characterization is also an important factor to be considered. The size, charge, surface modifications, stability, toxicity is closely related to the action of these particles in the body. The size, shape, charge, and capacity to incorporate therapeutic agents inside the nanocarrier make the nanoparticle system suitable for therapy. Currently, liposomes, PLGA (polylactic-co-glycolic acid) nanoparticles, dendrimers, gold nanoparticles, micelles, polymeric nanoparticles, and microneedles are widely studied for targeting particular immune cells or immune reactions. The combination of biomaterials and drugs using nanotechnology has resulted in interesting outcomes regarding the treatment strategies [15,16,17,18]. A number of biomaterials have been studied for targeting particular immune cells for specific immune reactions [19,20]. The application of biomaterials for immune treatment is being widely studied for many diseases, especially cancer therapy [21,22,23]. This review summarizes the mechanism of immune reactions against cancer and how immunotherapy works against cancer. Moreover, we discuss currently available bioactive nanoparticle systems for cancer immunotherapy and the mode of action of these nanoparticles against cancer.

## 2. Cancer Immune Response

Our health is maintained by the immune response as one of the key factors. The tumor immune response is complex; however, many studies have been conducted to discover the interesting mechanisms behind the response. As is known about innate and adaptive immunity, both of these immune responses together help the body maintain its equilibrium [24,25,26]. Discrimination of the immune system between the self and non-self allows the immune response easily detect and eliminate the unwanted or malignant cells from the body [27,28,29]. The first-line immune response of our body starts from the skin and mucous physical barriers to the complex T cell and B cell immune responses.

Both the innate and adaptive immune responses are different and complex in nature. The innate immune system mainly acts through the phagocytosis pathway, which is composed of phagocytic cells such as natural killer (NK) cells, macrophages, neutrophils, and monocytes. The main mode of action of the innate immune response is to discriminate the proteins presented by these cells from those of invading cells. The factors that are able to discriminate these presented proteins from those of the tumor cells are called major histocompatibility complex class I (MHC-I) [30,31,32,33,34]. The adaptive immune responses represent specific and effective methods to inhibit tumor growth. Tumor-associated antigens (TAAs) are the main prompting factor for immune reactions. TAAs can be recognized by both T cells and B cells; thus, targeting TAAs would be a better way to inhibit tumorigenesis. Targeting of TAAs can be done in many ways, among which targeting using antigens is more common and effective. Another method to inhibit tumor growth is to target the immune checkpoint with different specific antibodies [35,36,37,38,39,40].

**Figure 1:**
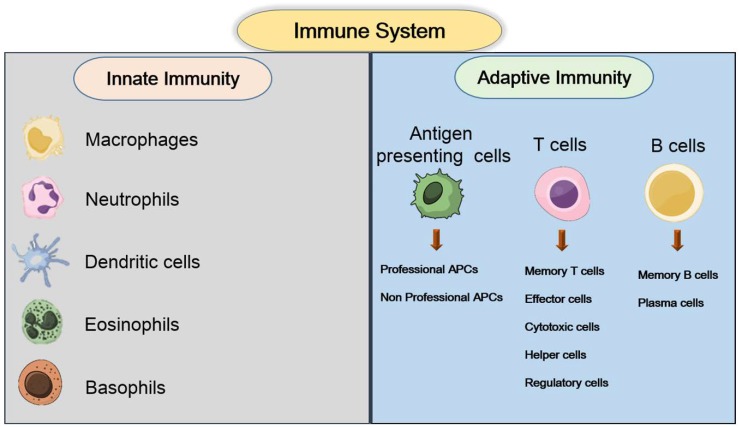
Cells and components involved in the innate and adaptive immune system.

Immune checkpoint blockade as a therapeutic modality that has gained more attention after the modification of CAR T cell-modified T cells to block specific immune regulatory checkpoints. The advancement in checkpoint blockade therapy resulted in the invention of the PD-1 (programmed cell death-1) and PDL-1 (programmed cell death ligand-1) blockade method, which is characterized by both the specificity and the improved outcome. Another intracellular protein present in T cells, cytotoxic T lymphocyte-associated protein-4, can also be utilized to inhibit the regulation of T cells followed by the arrest of both the proliferation and activation of the tumor cells [41,42,43,44,45,46]. The major cells and components involved in the innate and adaptive immune responses are given below (Figure 1) [47].

The mode of action of the immune response against tumors is based on how the immune system can differentiate between cancer cells and normal cells. The main difference between cancer cells and normal cells is the expression of various kinds of TAAs in cancer cells. The killing of cancer cells can be achieved by both immunogenic and tolerogenic signals. The immunogenic signals are released due to the rapid growth of the tumor followed by necrosis. The necrosis of the tumor will result in the release of TAAs and immunogenic signals followed by the activation of phagocytes and antigen-presenting cells (APCs). This kind of activation results in the production of cytokines such as interferon gamma (IFN-γ) and tumor necrosis factors, leading to the upregulation of MHC class I expression and tumor suppression. The tolerogenic signals can act in main three ways. The downregulation of MHC class I and killing of tumors by TAA-specific cytotoxic T lymphocytes (CTLs) is considered to be one way of killing tumors. In another way, CTL-mediated killing mechanisms can evade immune surveillance. Finally, tumor killing occurs by programmed cell death PD-L1 via activated T cells [48].

## 3. Cancer Immunotherapy

**Figure 2:**
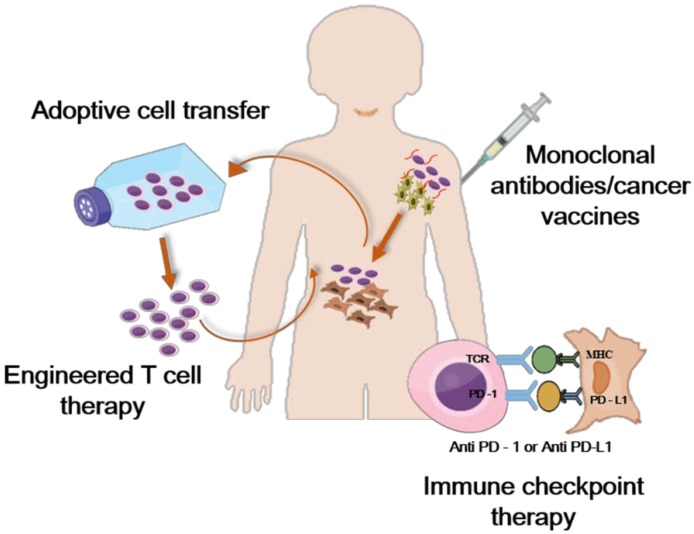
Types of immunotherapies and mechanism of programmed cell death. (Redrawn from Copy right © Ivyspring International Publisher. This is an open access article distributed under the terms of the Creative Commons Attribution (CC BY-NC) license (https://creativecommons.org/licenses/by-nc/4.0/)).

A number of cancer immunotherapeutic approaches have been explored widely. However, many new studies have utilized dendritic cell (DC) vaccines, immune checkpoint inhibitors, monoclonal antibodies, cytokines, and adoptive cell therapies [49]. The modes of immunotherapy can be divided into passive or active immunotherapy. The administration of monoclonal antibodies and cytokines is considered to be a passive mode of immunotherapy that is effective in activating the antitumor immune response in the body. DC vaccines, checkpoint inhibitors and adoptive T cell engineering and therapy are considered to be active modes of therapy; hence, they are used to stimulate our self-immune system to attack cancer. A brief idea about the types of immunotherapy and the mode of programmed cell death is given below (Figure 2) [50].

The efficacy and safety of this immunotherapy opened a new window for clinicians to explore and evolve for the betterment of cancer therapy. The discovery of immune checkpoint blockade and CAR T cells for inhibiting tumor growth led immunotherapy to be the suitable modality for cancer treatment. Newer monoclonal antibodies, targeting new immune system checkpoints, the invention of tremendous cancer vaccines and oncolytic viruses, the modification of CAR T cells and the discovery of tumor-infiltrating lymphocytes and interleukin-2 make this area of treatment different from other treatment methods [51,52,53].

## 4. Bioactive Nanoparticles for Cancer Immunotherapy

Bioactive nanoparticles are considered to be the prime option for immunotherapy due to their special characterizations, such as size, shape, charge, elasticity, and ability to work as a carrier [54,55]. Nanoparticle engineering could be useful for the modification of nanoparticles, particularly for targeting, carrying antigens/adjuvants or drugs for immunotherapy. The size of the nanoparticle can affect the pharmacokinetics, transport, and cellular uptake of the nanoparticle, which can critically affect the therapeutic efficacy [56]. The shape of the nanoparticle also greatly affects the circulation and accumulation of the nanoparticle in the desired site [57].

**Figure 3:**
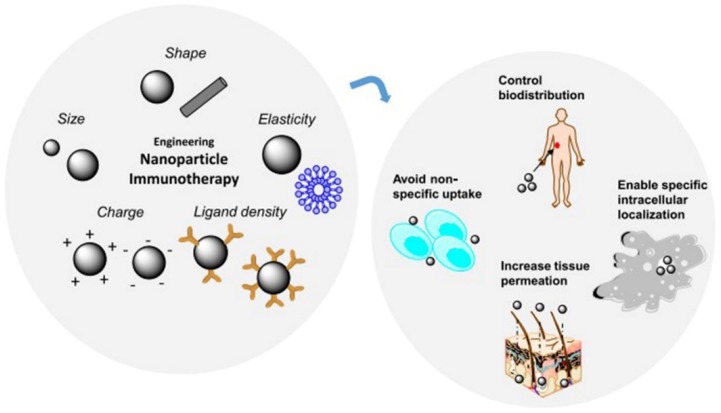
Possible nanoparticle engineering for cancer immunotherapy. (Copy right © 2016 The Authors. Bioengineering & Translational Medicine is published by Wiley Periodicals, Inc. on behalf of The American Institute of Chemical Engineers. This is an open access article under the terms of the Creative Commons Attribution License, which permits use, distribution and reproduction in any medium, provided the original work is properly cited.).

Another major factor that affects the internalization of nanoparticles into cells is the charge of the nanoparticles. The surface modification of nanoparticles can be accomplished by applying various surface chemistries. Cationic particles accumulate more in tumor cells. Modification of a nanoparticle surface to obtain a positive charge can be utilized. Hence, cationic particles show higher toxicity, and advancements in modification are currently being investigated by many researchers. These three modifications are the major ones to be considered before synthesis of a nanocarrier or nanoparticle for immunotherapeutic use. Other than these modifications, the nanoparticle ligand density and nanoparticle elasticity can also be tuned for improved transport and accumulation of the nanoparticles (Figure 3) [58]. These modifications will enable nanoparticles to increase tumor accumulation and localization and to avoid target uptake.

**Figure 4:**
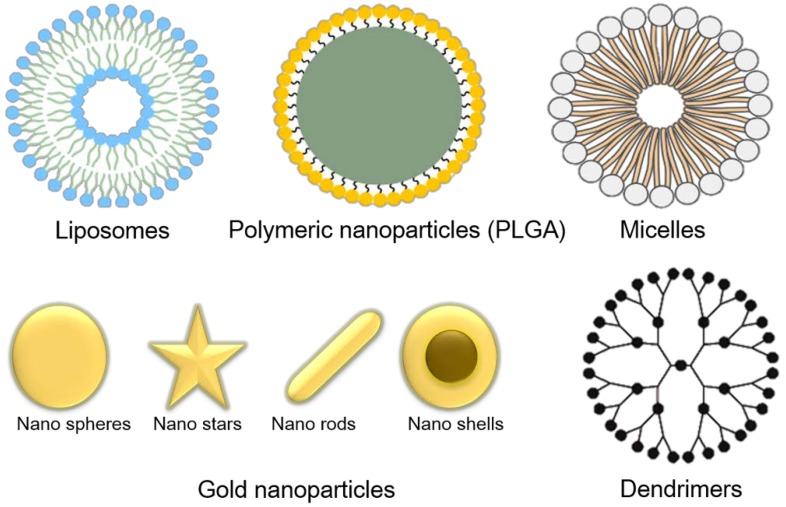
Different nanoparticle systems currently used for cancer immunotherapy.

The various bioactive nanoparticles enable them to be the primary option for immunotherapy (Figure 4). The application of these bioactive nanoparticles in cancer therapy has been studied for decades. Among a number of bioactive nanoparticle systems for cancer immunotherapy, polymeric nanoparticles are the most common. PLGA is one of the most studied FDA-approved polymeric carriers for cancer immunotherapy due to its nontoxicity and biodegradability [59,60,61]. In addition to PLGA lipids, PEG (polyethylene glycol) is also commonly used for the synthesis of nanocarrier systems as vehicles for immunotherapy. Liposomes, micelles, dendrimers, inorganic nanoparticles such as gold nanoparticles, iron oxide nanoparticles, carbon nanoparticles, and quantum dots are also commonly used as carriers due their specific targeting, nontoxicity, and biodegradability [62,63,64]. All of these nanoparticles can be utilized to track cancer and treat tumors with minimal off targeting. The final goal of bioactive nanoparticles is to successfully deliver antigens, adjuvants or other immunotherapeutic agents to the desired target sites, such as lymph nodes or other intracellular locations, for the activation of the immune response.

### 4.1. PLGA Nanoparticles

PLGA is considered to be an excellent component for immunotherapy due to its low systemic toxicity and high biodegradability compared to other polymeric systems. The majority of PLGA-based nanoparticle studies for cancer immunotherapy are based on targeting the nanoparticle system to DCs. PLGA nanoparticles are taken up by DCs without any recognition of this specific character [65,66]. Targeting DCs using PLGA nanoparticles for the delivery of antigens, vaccines and other therapeutic moieties for immunotherapy is widely studied. One study shows that PLGA nanoparticles are more capable of targeting DCs than PLGA microparticles are. The in vitro results exhibited a lower delivery of humanized targeting antibody (hD1) to the targeted DC cells when microparticles were used. At the same time, nanoparticles showed a 10- to 100-fold higher efficiency in delivery of hD1 [67]. Modification of the PLGA nanoparticle surface for better targeting to DCs has been studied to increase the delivery of more antigens to the desired site. Modification of the surface of the nanoparticle with maximum densities of monoclonal antibody (mAb) to target the cluster of differentiation -205 (DEC-205) receptor resulted in DC immunization with higher interleukin-10 (IL10) production [68].

The antitumor cytotoxic T cell response can be induced by the delivery of vaccines to the DC by targeting DCs using PLGA nanoparticles. CD40-mediated delivery of vaccines to DC cells using PLGA nanoparticles resulted in an antitumor response with prolonged survival. This study reveals that PLGA nanoparticles could be a perfect option for targeting DCs to deliver vaccines [69]. PLGA nanoparticles containing antigenic peptides were successfully delivered to the DCs, followed by the cytotoxic T cell immune response both in vitro and in vivo. These nanoparticles could be more useful to block the immune escape mechanism of tumor cells [70].

The combination of PD-1 blockade and laser immunotherapy can also be achieved by PLGA nanoparticles. After the intratumoral injection of gold nanoshells and anti PD-1 peptide (APP)-loaded PLGA nanoparticles, an excellent killing effect at the primary tumor site was achieved by photothermal therapy (PTT). These nanoparticles also played a vaccine-like role and induced a localized antitumor-immune response. APP release with PTT transient triggering could induce the blockage of the PD-1/PD-L1 pathway to activate T cells, thus subsequently generating a systemic immune response. This therapeutic platform could efficiently kill primary cancers and treat the growth of metastatic tumors [71].

### 4.2. Liposomes

Liposomes for the delivery of cancer immunotherapeutic agents are widely studied among different lipid-based nanoparticles. Co-delivery of ovalbumin (OVA) and IFN-γ-encoding pDNA to the DCs by utilizing pH-sensitive liposomes was found to be an effective treatment option. The combined therapeutic effect of OVA and IFN-γ-encoding pDNA in tumor-bearing mice promoted the infiltration of CTLs into the tumor and resulted in a strong antitumor effect [72]. Antigenic peptides (OVA) encapsulated with pH-sensitive fusogenic polymer-modified liposomes showed a good antitumor effect in the OVA-expressing OVA tumor-bearing mice. The CTL activation was found to be much higher than in the control groups. These liposomes might be useful for improving CTL-inducing peptides for efficient cancer immunotherapy [73].

Recently, Yuba E et al. studied the strong immunotherapeutic effect of pH-sensitive dextran liposomes modified with PEG as well as TGF-β 1 (transforming growth factor) receptors. The liposome delivery resulted in a high antitumor effect by the infiltration of CD8-positive cells to the tumor [74]. Yuba et al. reported that liposomes modified with pH-sensitive polymers such as curdlan and mannan were used as bioactive polysaccharides. These pH-sensitive liposomes released their contents at weakly acidic pH and delivered model antigenic proteins into the cytosol of DCs. Subcutaneous injection of these liposomes induced strong antigen-specific immune responses and stronger antitumor effects than those of liposomes modified with a dextran derivative [75].

To overcome the immunosuppressive tumor microenvironment (TME), it is very important to enhance the efficacy of cancer immunotherapy. Agonists of stimulator of interferon genes (STING), a cytosolic immune adaptor protein, have been shown to induce potent antitumor activity when delivered into the TME. The anionic properties of STING agonists make them poorly membrane permeable, cationic liposomes with varying surface polyethylene glycol levels, and they can be used to encapsulate 2′3′-cyclic guanosine monophosphate-adenosine monophosphate (cGAMP) to facilitate its cytosolic delivery. Liposomal delivery improves STING agonist activity and results in immunological memory that helps rechallenge tumor cells [76].

Another area of immunotherapy by gene delivery also utilizes liposomes as the carrier for specific targeting. The delivery and targeting of RNA to DC cells using lipoplexes also has gained more attention in cancer immunotherapy. The delivery of RNA lipoplex (RNA-LPX) to DC cells activates INF-α-mediated immune mechanisms, resulting in in situ DC maturation followed by innate immune reactions. The results were promising for cancer immunotherapy; therefore, RNA-PLX systems can be utilized as a better therapeutic agent for DC targeting [77].

Generally, PEGylation is commonly used in these kinds of studies to modify lipid nanoparticles for the delivery of siRNA. A pH-sensitive cationic lipid called YSK05 for the formulation of a multifunctional envelope-type nanodevice (MEND) is used as a carrier for siRNA to overcome the limitations followed by optimization of the lipid composition. The intratumoral administration of the PEGylated YSK05-MEND enhanced gene silencing and was found to be a good agent for delivery of siRNA into the cytosol [78].

### 4.3. Micelles

The use of micelles for cancer therapy has been studied and explored both preclinically and clinically. There is a wide range of applications of micelles in cancer treatment as carriers for imaging, chemotherapy, radiotherapy, and immunotherapy. The synthesis of micelles is comparatively easier than that of other nanoparticles. The biodegradability and nontoxicity of these formulations make them suitable for carrying therapeutic payloads [79]. Antigen delivery to the cytoplasm usually uses pH-responsive liposomes or other kinds of liposomes. However, new studies show that the cytoplasmic delivery of antigens is possible using micelles as carriers. A pH-responsive micelle composed of dilauroyl phosphatidylcholine (DLPC) and deoxycholic acid was synthesized both to deliver antigens to the cytoplasm and to induce an immune response. Micelles were taken up by DCs mainly via macropinocytosis and delivered OVA into the cytosol. These micelles are useful for increasing the capability of cellular immunity in the treatment of cancer [80].

Another study showed the stimulation of the immune response using micelles with the combined action of PTT with immunotherapy. The immune response can be stimulated by regulating metabolism-related enzymes. Due to the accumulation of IR780 in the tumor followed by migration to the lymph node, PTT can be performed, resulting in the inhibition of IDO (indoleamine 2,3-dioxygenase). The inhibition of IDO leads to the activation of T lymphocytes followed by the inhibition of distal tumor growth (abscopal effect). Combined therapy of this micellar system kills the tumor by PTT and inhibits distal tumor growth post-PTT in vivo in BALB/c-nu mice [81].

Polymeric micelles are currently considered to be a more exploratory nanoparticle carrier system for cancer immunotherapy. The delivery of tyrosinase-related protein 2 peptide antigen and adjuvant to the lymph node in the B16F0 melanoma mouse model was performed using cationic diblock polymeric micelles. The resulting increased T lymphocyte anticancer activity indicates the efficiency of these kinds of micelles in treating cancer by improving the immune response [82].

Repolarization of Tumor associated macrophages (TAMs) to M1 macrophages can be used as a strategy for cancer immunotherapy. The use of galactose-functionalized zinc protoporphyrin IX grafted polypeptide micelles for targeting TAMs and delivering immunopotentiators resulted in the induction of ROS and decreased STAT3 expression. Activation of T lymphocytes by the repolarization of TAMs resulted in tumor regression [83].

### 4.4. Gold Nanoparticles

**Figure 5:**
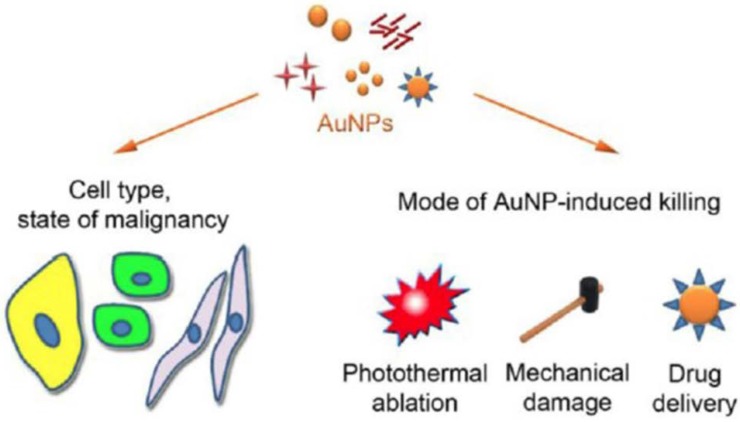
Overall scheme of different gold nanoparticles for cancer immunotherapy and their mode of therapeutic action in different immune cells. (Copy right © Ivyspring International Publisher. This is an open access article distributed under the terms of the Creative Commons Attribution (CC BY-NC) license (https://creativecommons.org/licenses/by-nc/4.0/)).

Gold nanoparticles for immunotherapy are considered to be a highly promising area of research due to their favorable characteristics. Recently, a number of studies have indicated that the ability of gold nanoparticles makes them a perfect carrier for immunotherapy [84]. These nanoparticle systems are broadly used as carriers for antigenic proteins and gene/oligonucleotide delivery to specific sites of interest. At the same time, research on combination therapy with PTT has also been in progress [85,86,87,88]. Different kinds of gold nanoparticles have been synthesized and studied as therapeutic carriers for cancer treatment. The type of gold nanoparticles, including their size, charge, shape, and functional group, has also contributed to the efficacy in accumulating different immune cells (Figure 5) [89].

Delivery of antigens using different gold nanoparticles is possible due to their high affinity towards antigens; at the same time, the affinity depends on the size and shape of the gold nanoparticles that are used. Delivery of CpG for immunotherapy was attempted in different sized and shaped gold nanoparticles, such as gold nanorods, nanoshells, and nanostars of different sizes. The size of the nanoparticle also plays a very important role in the delivery of antigens to the desired site. It has been observed that the 50- and 15-nm gold nanospheres are the perfect option for immunotherapeutic delivery of antigens [90].

Delivery of antigens/adjuvants such as OVA and CpG using gold nanoparticles for the in vivo B16-OVA tumor model is an effective method to treat cancer by activating the immune system. The results of this study showed an antigen-specific immune response that led to an antitumor response and an improved survival rate [91]. Delivery of the CpG oligonucleotide immunostimulant using gold nanoparticles resulted in better accumulation of the nanoparticle. These gold nanoparticles induced the infiltration of macrophages and DCs, leading to the regression of tumor growth. The study was proved both in vitro and in vivo, suggesting that the use of gold nanoparticles could be a good method for antigen delivery [92]. Currently, gold nanoparticles have also been used for image-guided immune checkpoint blockade. Gold nanoparticles conjugated to α-PDL1 showed image-guided tracking and therapeutic effects [93].

Gold nanoparticles can be used both as potent carriers for therapeutic moieties and as PTT agents. The combined PTT and immunotherapeutic effect of gold is possible due to its optical and therapeutic characteristics. The PTT effect resulted in the expression of cytokines and chemokines followed by DC maturation and the T cell immune response. Gold nanoshells could be used both as a PTT agent and an effective immunotherapeutic agent [94]. Another group utilized gold nanoshells for a different kind of immune responses by activating inflammasome complexes. Thermal ablation results in the activation of inflammasomes, resulting in an immunotherapeutic effect by activating proinflammatory cytokines in vivo [95]. Combination therapy was successfully performed by the delivery of gold nanoparticles for PTT therapy and tumor necrosis factor-α (TNF-α) for immunotherapy. The combined effect of both TNF-α and the PTT effect of gold exhibited an improved antitumor effect in vivo in SCK tumors [96].

### 4.5. Iron Oxide Nanoparticles 

Iron oxide nanoparticles can also be used as potent carriers for vaccine delivery and can be used as antitumor agents for cancer therapy. A very recent study on iron oxide nanoparticles for vaccine delivery showed an improved therapeutic effect. In this study, the authors used a superparamagnetic Fe_3_O_4_ as a delivery system of OVA and as an immune potentiator. The delivery of iron oxide nanoparticles alone exhibited both immune cell activation and cytokine production. These results confirm the immunotherapeutic effect of iron oxide nanoparticles by themselves in a colon adenocarcinoma (CT26) tumor animal model [97]. Another mode of action of iron oxide nanoparticles in cancer immunotherapy is by polarizing immune cells such as DCs and macrophages. This polarization of immune cells will result in an increased immune response against tumors. The administration of FDA-approved ferumoxytol iron supplementation in a mammary cancer model displayed macrophage polarization and increased caspase-3 activity [98]. Other groups have studied DC polarization by the action of branched polyethylenimine–superparamagnetic iron oxide nanoparticles. UVB irradiation in the tumor cells resulted in apoptosis and subsequent tumor antigen production and antitumor immune response. The Th1 polarization of DCs after treatment with iron oxide nanoparticles demonstrated the therapeutic ability of these nanoparticles in treating cancer [99].

### 4.6. Others

Dendrimers are used to deliver OVA to immune cells by incorporating OVA into guanidine-terminated dendrimers by utilizing the helix B region of OVA [100]. Immunodendrimers are used to treat ovarian cancer in BALB/c mice. A half-generation poly(propyl imine) dendrimer is conjugated with an immunotherapeutic antibody and loaded with the anticancer drug paclitaxel. This immunodendrimer significantly reduced systemic toxicity and tumor volume, demonstrating the efficacy of dendrimers as a carrier vehicle for both therapeutic drug and antigens [101].

Exosomes are basically secreted by immune cells, and they are characterized to deliver proteins and antigens for therapy. The use of biomimetic exosomes for the delivery of cargos for immunotherapy has gained more attention. Delivery of the monoclonal antibody DEC205 to dendritic cells can be performed using biomimetic exosomes. The synthesis of these exosomes is similar to liposomes. The study was a novel method of delivering antigens to DCs for immunotherapy [102]. The synthesis of artificial exosomes is very easy compared to other carrier systems, and this method of treatment could be a key to future nanomedicine for immunotherapy. Artificial exosomes can be modified using MHC class I peptides and liposomes for DC targeting and T cell activation. The in vivo studies showed increased T cell activation by the action of MHC class I peptide delivery to the DCs. The liposome peptide containing artificial exosomes was found to be very stable and suitable for targeting DCs [103]. Types of nanoparticles used for cancer immunotherapy is given below (Table 1).

## 5. Combinational Immunotherapy

Among the number of treatment modes for cancer therapy, immunotherapy was found to be more effective with better outcomes. Currently, there are numerous studies being published that combine immunotherapy with other modes of therapy, such as PTT, photodynamic therapies (PDTs), radiotherapy, and chemotherapy. Utilizing immunotherapy with other therapy combinations appears to be a potent way to eradicate cancer and will result in more specific and effective antitumor effects. Overall, combination therapy has resulted in a complete antitumor effect with less risk of tumor recurrence and no metastatic progression. Combination therapy could be a milestone future cancer therapy with low side effects and good results.

There are many studies in which PTT and immunotherapy combinations are used to eliminate cancer. The major benefit of combining PTT with immunotherapy using nanoparticles is the relatively lower risk of tumor recurrence [104]. PDT in combination with immunotherapy is also being studied. Song et al. used a nanoparticle that was synthesized from chimeric peptides and consisted of a photosensitizer PpIX with an immune checkpoint inhibitor called 1MT. The nanoparticle generates ROS upon 630 nm light irradiation, leading to necrosis followed by caspase-3 expression and tumor antigen production. The synergistic effects kill both primary and lung malignancies effectively [105].

The combination of immunotherapy with radiotherapy can also be utilized to obtain an improved antitumor effect in different cancer models; hence, the possibility of tumor antigen release is greater after radiation treatment. A plant-based virus-like (VLP) nanoparticle was used for the delivery of radiation therapy in patients with oral melanoma. VLP nanoparticles increased the infiltration of immune cells to the tumor site, followed by an immune response and tumor killing after radiation [106].

Another area of combinational therapy that utilizes chemotherapy and immunotherapy has also gained more attention in cancer therapy. Numerous studies have also been performed based on this strategy to completely eradicate cancer and reduce the risk of recurrence. Immunosuppressive tumor microenvironment (TME)-responsive nanocarriers were used to deliver PTX, mitoxantrone (MIT) and celastrol (CEL). The importance of delivering drugs is to induce both chemotherapeutic and immunotherapeutic effects synergistically. This combined action of drugs resulted in a better antitumor effect and prevented metastatic progression [107].

## 6. Conclusions

Immunotherapy is one of the most explored areas in the new era of cancer treatment due to its specificity and fewer side effects compared to the other modality of therapy for cancer. A number of methods have been explored to generate an immune response against cancer by developing new immunotherapeutic agents through the modification of current treatment agents and the development of new carriers for improved targeting. These approaches can be considered a milestone in this area of cancer treatment.

A number of different bioactive materials have been studied preclinically and clinically for cancer therapy. The use of biomaterials in the form of nanoparticles also increased the effectiveness of the therapy due to the special characteristics of these nanoparticle systems. The specificity and targeting ability of the nanoparticle can be altered using different therapeutic moieties, polymers, and targeting agents. Liposomes, PLGA nanoparticles, micelles, dendrimers, and inorganic nanoparticles are commonly used bioactive nanoparticle systems for cancer immunotherapy. All of these nanoparticle systems are currently studied widely, and research is ongoing. The advantages of these kind of bioactive systems are their high targeting ability, low toxicity, biodegradability, and high specificity. However cost for cancer immunotherapy also considerably high when it comes to the clinical perspective. The increased cost of nanomedicine limits this therapeutic options to be in mainstreams. However new approaches are coming out to make cost effective nanoparticle systems for future cancer immunotherapy. The major factors which is to be considered in a better clinical outcome would be the efficiency, potency, toxicity, and cost effectiveness of the nanoparticle systems.

Delivering antigens/adjuvants or other drugs/vaccines to the desired cells or lymph nodes can be done effectively by the use of these types of nanovehicles and is accomplished more easily than other methods of immunotherapy. Liposomes for cancer treatment are currently being studied at the clinical level and represent a potent carrier system for cancer therapy. There are a number of drugs are being studied clinically for cancer immunotherapy such as atezolizumab, nivolumab, pidilizumab, paclitaxel, etc. The major mechanism of action of these drugs are anti-PD1 as well as anti-PDL1. Some of these drugs are currently in clinical trial III phase and some are in phase I and II. To date, a number of studies have indicated that bioactive nanoparticle-assisted immunotherapy could be a perfect tool for future cancer treatment. All these kind of nanoparticles exhibits some unique characteristics which makes them suitable candidate for immunotherapy. In the case of liposomes, it can load both hydrophilic and hydrophobic moieties inside, which enable the delivery of different kinds of therapeutics into the tumor site. PLGA nanoparticles are food and drug administration (FDA) approved and widely used in clinical field for controlled drug delivery as well as in manufacturing of surgical sutures. At the same time PLGA nanoparticles can be used to target specially to DCs which can be utilized for immunotherapy. In the case of micelles, a number of polymeric moieties can be used to synthesize different types of micelles. These polymers can be responsible for a number of immune response which helps in cancer immunotherapy. Moreover the release of therapeutics from micelles can be facilitated by different stimuli responses which can be utilized in immunotherapy. The advantage of use of gold nanoparticles for immunotherapy is wide ranging from their optical properties to therapeutic effect. It can be used for imaging, deliver of macromolecules for therapy and also for combination therapy such as thermal ablation. All these bioactive nanoparticles are unique and suitable for immunotherapy for cancer. Combined immunotherapy with other treatment methods has also recently gained attention. This combination of PTT, chemotherapy, and radiotherapy with immunotherapy can hopefully contribute to future cancer treatment.

## Figures and Tables

**Table 1:** Types of nanoparticle systems used for immunotherapy, containing therapeutic agents, and their functions in different tumor models.

Material	Therapeutic Agents	Target	Function	Tumor Model	Size, Charge and Polydispersity Index	Surface Modifications	Ref.
PLGA based nanoparticles	AUNP12 anti-PD-1 peptide	Tumor cells	Blockage of PD-1/PDL-1 Pathway	4T1 Subcutaneous tumor	400–600 nm, positive charge	-	[71]
	Trastuzumb	Human epidermal growth factor 2 (HER2)	HER2 degradation and antibody-dependent cell-mediated cytotoxicity	*Invitro* HER2 positive breast cancer model	174 ± 43.9 nm, −21.7 ± 8.6 mV and 0.138 ± 0.066 PDI	-	[65]
	Pam3CSK4 and α-CD40-mAb	CD40	T cell response	B16-OVA Subcutaneous tumor	209.8 ± 11.1 nm, −32.2 ± 2.8 mV and 0.114 ± 0.022 PDI	Coating with agonistic α-CD40-mAb	[69]
Liposomes	SB505124 TGF-β 1 inhibitor	Tumor specific cytotoxic T-lymphocyte CTLs	Block TGF-β Signal and promote CD8+ T cell infiltration	E.G7-OVA Subcutaneous tumor	114 ± 15 nm	3-Methylglutarylated dextran (MGlu-Dex)-modified liposomes	[74]
	Curdlan and mannan	Cytosol of DCs	Activation of DCs via Th1 cytokine production	DC2.4 in vitro model	100–157 nm, negative charge	Polysaccharide derivative modified liposomes	[75]
	Stimulator of interferon genes (STING) agonists and cGAMP	Tumor microenvironment (TME)	Pro-inflammatory gene induction and production of immunological memory	B16-F10 Lung metastatic tumor	160 nm and 42 mV	-	[76]
Micelles	Pyranine antigen	Cytoplasm of DCs	Antigen specific cellular immunity	C57BL/6 intradermal immunized mice	12 nm and −30 mV	-	[80]
	NLG919/IR780	Lymph node	Suppression of growth of tumor margin in primary tumors	4T1 Subcutaneous tumor	43 ± 3.2 nm	-	[81]
	ROS inducing ZnPP PM/PIC	Tumor associated macrophages (TAMs)	Activation of NK cells and T lymphocytes	B16-F10 Subcutaneous tumor	75–82nm, −10–18 mV and 0.2 PDI	-	[82]
Gold nanoparticles	OVA peptide antigen/CpG adjuvant	Dendritic cells	Induce systemic antigen specific immune response	B16-OVA Subcutaneous tumor	15–80 nm	-	[92]
	α-PDL1	Tumor cells	Imaging and tumor reduction	Colon cancer subcutaneous tumor	20 nm	α-PDL1 conjugation	[93]
Iron oxide nanoparticles	Superparamagnetic Fe_3_O_4_	DCs and macrophages	Immune cell activation and cytokine production	CT2 Subcutaneous tunor	600–900 nm, −20–25 mV	OVA conjugation	[97]
	Ferumoxytol	Macrophages	Increased caspase-3 activity and pro-inflammatory Th1 response	MMTV-PyMT Mammary tumor	-	-	[98]
Dendrimers	mAbK1/PTX	Tumor cells—mesothelin receptors	Specific binding and anti-tumor activity	OVCAR3 Subcutaneous tumor	-	surface modification using AbK1	[101]
Artificial exosomes	DEC205 monoclonal antibody	Dendritic cells	Targeting to DCs	In vitro studies-DCs	100 nm	MHC Class I peptide surface coating	[103]

## References

[1] Farkona S., Diamandis E.P., Blasutig I.M. (2016). Cancer immunotherapy: The beginning of the end of cancer?. BMC Med..

[2] Kokate R. (2017). A Systematic Overview of Cancer Immunotherapy: An Emerging Therapy. Pharm. Pharmacol. Int. J..

[3] Neves H., Kwok H.F. (2015). Recent advances in the field of anti-cancer immunotherapy. BBA Clin..

[4] McCune J.S. (2018). Rapid Advances in Immunotherapy to Treat Cancer. Clin. Pharmacol. Ther..

[5] Sun W. (2017). Recent advances in cancer immunotherapy. J. Hematol. Oncol..

[6] Androulla M.N., Lefkothea P.C. (2018). CAR T-cell Therapy: A New Era in Cancer Immunotherapy. Curr. Pharm. Biotechnol..

[7] (2017). Engineering CAR T Cells with Biomaterials. Cancer Discov..

[8] Hargadon K.M., Johnson C.E., Williams C.J. (2018). Immune checkpoint blockade therapy for cancer: An overview of FDA-approved immune checkpoint inhibitors. Int. Immunopharmacol..

[9] Wieder T., Eigentler T., Brenner E., Röcken M. (2018). Immune checkpoint blockade therapy. J. Allergy Clin. Immunol..

[10] Kroschinsky F., Stölzel F., von Bonin S., Beutel G., Kochanek M., Kiehl M., Schellongowski P. (2017). New drugs, new toxicities: Severe side effects of modern targeted and immunotherapy of cancer and their management. Crit. Care.

[11] Yang L., Yu H., Dong S., Zhong Y., Hu S. (2017). Recognizing and managing on toxicities in cancer immunotherapy. Tumor Biol..

[12] Park W., Heo Y., Han D.K. (2018). New opportunities for nanoparticles in cancer immunotherapy. Biomater. Res..

[13] Velpurisiva P., Gad A., Piel B., Jadia R., Rai P. (2017). Nanoparticle Design Strategies for Effective Cancer Immunotherapy. J. Biomed..

[14] Zang X., Zhao X., Hu H., Qiao M., Deng Y., Chen D. (2017). Nanoparticles for tumor immunotherapy. Eur. J. Pharm. Biopharm..

[15] Yu Y., Cui J. (2018). Present and future of cancer immunotherapy: A tumor microenvironmental perspective (Review). Oncol. Lett..

[16] Chen D.S., Mellman I. (2013). Oncology meets immunology: The cancer-immunity cycle. Immunity.

[17] Oiseth S.J., Aziz M.S. (2017). Cancer immunotherapy: A brief review of the history, possibilities, and challenges ahead. J. Cancer Metastasis Treat..

[18] Martin-Liberal J., Ochoa de Olza M., Hierro C., Gros A., Rodon J., Tabernero J. (2017). The expanding role of immunotherapy. Cancer Treat. Rev..

[19] Rajendrakumar S.K., Mohapatra A., Singh B., Revuri V., Lee Y.K., Kim C.S., Cho C.S., Park I.K. (2018). Self-assembled, adjuvant/antigen-based nanovaccine mediates anti-tumor immune response against melanoma tumor. Polymers.

[20] Rajendrakumar S.K., Uthaman S., Cho C.S., Park I.K. (2018). Nanoparticle-Based Phototriggered Cancer Immunotherapy and Its Domino Effect in the Tumor Microenvironment. Biomacromolecules.

[21] Zhang R., Billingsley M.M., Mitchell M.J. (2018). Biomaterials for vaccine-based cancer immunotherapy. J. Control. Release.

[22] Hu Z., Ott P.A., Wu C.J. (2018). Towards personalized, tumour-specific, therapeutic vaccines for cancer. Nat. Rev. Immunol..

[23] Wang C., Ye Y., Hu Q., Bellotti A., Gu Z. (2017). Tailoring Biomaterials for Cancer Immunotherapy: Emerging Trends and Future Outlook. Adv. Mater..

[24] Dougan M., Dranoff G. (2009). The immune response to tumors. Curr. Protoc. Immunol..

[25] Spranger S., Gajewski T.F. (2018). Impact of oncogenic pathways on evasion of antitumour immune responses. Nat. Rev. Cancer.

[26] Pandolfi F., Cianci R., Pagliari D., Casciano F., Bagal C., Astone A., Landolfi A., Barone C. (2011). The immune response to tumors as a tool toward immunotherapy. Clin. Dev. Immunol..

[27] Gonzalez S., González-Rodríguez A.P., Suárez-Álvarez B., López-Soto A., Huergo-Zapico L., Lopez-Larrea C. (2011). Conceptual aspects of self and nonself discrimination. Immune Recognit. Signal..

[28] Paul W.E. (2010). Self/nonself-immune recognition and signaling: A new journal tackles a problem at the center of immunological science. Immune Recognit. Signal..

[29] Tanasescu R., Constantinescu C.S. (2010). Cannabinoids and the immune system: An overview. Immunobiology.

[30] Berraondo P., Minute L., Ajona D., Corrales L., Melero I., Pio R. (2016). Innate immune mediators in cancer: Between defense and resistance. Immunol. Rev..

[31] O’Sullivan T., Saddawi-Konefka R., Vermi W., Koebel C.M., Arthur C., White J.M., Uppaluri R., Andrews D.M., Ngiow S.F., Teng M.W. (2012). Cancer immunoediting by the innate immune system in the absence of adaptive immunity. J. Exp. Med..

[32] Messerschmidt J.L., Prendergast G.C., Messerschmidt G.L. (2016). How Cancers Escape Immune Destruction and Mechanisms of Action for the New Significantly Active Immune Therapies: Helping Nonimmunologists Decipher Recent Advances. Oncologist.

[33] Hagerling C., Casbon A.J., Werb Z. (2015). Balancing the innate immune system in tumor development. Trends Cell Biol..

[34] Moynihan K.D., Irvine D.J. (2017). Roles for innate immunity in combination immunotherapies. Cancer Res..

[35] Tsai H.F., Hsu P.N. (2017). Cancer immunotherapy by targeting immune checkpoints: Mechanism of T cell dysfunction in cancer immunity and new therapeutic targets John T Kung. J. Biomed. Sci..

[36] Scott A.M., Renner C. (2001). Tumour Antigens Recognized by Antibodies. eLS.

[37] Wang Y., Wang X., Ferrone C.R., Schwab J.H., Ferrone S. (2015). Intracellular antigens as targets for antibody based immunotherapy of malignant diseases. Mol. Oncol..

[38] Michaud H.-A., Eliaou J.-F., Lafont V., Bonnefoy N., Gros L. (2014). Tumor antigen-targeting monoclonal antibody-based immunotherapy: Orchestrating combined strategies for the development of long-term antitumor immunity. Oncoimmunology.

[39] Trenevska I., Li D., Banham A.H. (2017). Therapeutic antibodies against intracellular tumor antigens. Front. Immunol..

[40] Spurrell E.L., Lockley M. (2014). Adaptive immunity in cancer immunology and therapeutics. Ecancermedicalscience.

[41] Korman A.J., Peggs K.S., Allison J.P. (2006). Checkpoint Blockade in Cancer Immunotherapy. Adv. Immunol..

[42] Ribas A., Wolchok J.D. (2018). Cancer immunotherapy using checkpoint blockade. Science.

[43] Buchbinder E.I., Desai A. (2016). CTLA-4 and PD-1 pathways similarities, differences, and implications of their inhibition. Am. J. Clin. Oncol. Cancer Clin. Trials.

[44] Gong J., Chehrazi-Raffle A., Reddi S., Salgia R. (2018). Development of PD-1 and PD-L1 inhibitors as a form of cancer immunotherapy: A comprehensive review of registration trials and future considerations. J. Immunother. Cancer.

[45] Shen X., Zhao B. (2018). Efficacy of PD-1 or PD-L1 inhibitors and PD-L1 expression status in cancer: Meta-analysis. BMJ.

[46] Zitvogel L., Kroemer G. (2012). Targeting PD-1/PD-L1 interactions for cancer immunotherapy. Oncoimmunology.

[47] Pandya P.H., Murray M.E., Pollok K.E., Renbarger J.L. (2016). The Immune System in Cancer Pathogenesis: Potential Therapeutic Approaches. J. Immunol. Res..

[48] Shao K., Singha S., Clemente-Casares X., Tsai S., Yang Y., Santamaria P. (2015). Nanoparticle-Based Immunotherapy for Cancer. ACS Nano.

[49] Disis M.L. (2014). Mechanism of action of immunotherapy. Semin. Oncol..

[50] Zhang H., Chen J. (2018). Current status and future directions of cancer immunotherapy. J. Cancer.

[51] D’Aloia M.M., Zizzari I.G., Sacchetti B., Pierelli L., Alimandi M. (2018). CAR-T cells: The long and winding road to solid tumors review-article. Cell Death Dis..

[52] Wei S.C., Duffy C.R., Allison J.P. (2018). Fundamental mechanisms of immune checkpoint blockade therapy. Cancer Discov..

[53] Labanieh L., Majzner R.G., Mackall C.L. (2018). Programming CAR-T cells to kill cancer. Nat. Biomed. Eng..

[54] Goldberg M.S. (2015). Immunoengineering: How nanotechnology can enhance cancer immunotherapy. Cell.

[55] Fan Y., Moon J. (2015). Nanoparticle Drug Delivery Systems Designed to Improve Cancer Vaccines and Immunotherapy. Vaccines.

[56] Chithrani B.D., Ghazani A.A., Chan W.C.W. (2006). Determining the size and shape dependence of gold nanoparticle uptake into mammalian cells. Nano Lett..

[57] Agarwal R., Singh V., Jurney P., Shi L., Sreenivasan S.V., Roy K. (2013). Mammalian cells preferentially internalize hydrogel nanodiscs over nanorods and use shape-specific uptake mechanisms. Proc. Natl. Acad. Sci. USA.

[58] Toy R., Roy K. (2016). Engineering nanoparticles to overcome barriers to immunotherapy. Bioeng. Transl. Med..

[59] Singh M.S., Bhaskar S. (2014). Nanocarrier-based immunotherapy in cancer management and research. ImmunoTargets Ther..

[60] Bookstaver M.L., Tsai S.J., Bromberg J.S., Jewell C.M. (2017). Improving Vaccine and Immunotherapy Design Using Biomaterials. Trends Immunol..

[61] Vranic E., Rahic O., Hadžiabdić J., Boskovic D. (2015). Opportunities and challenges for utilization of nanoparticles as bioactive drug carriers for the targeted treatment of cancer. Folia Med.—Fac. Med. Univ. Saraeviensis.

[62] Jia Y., Omri A., Krishnan L., McCluskie M.J. (2017). Potential applications of nanoparticles in cancer immunotherapy. Hum. Vaccin. Immunother..

[63] Fogli S., Paccosi S., Michelucci E., Berti D., Parenti A. (2017). Inorganic nanoparticles as potential regulators of immune response in dendritic cells. Nanomedicine.

[64] Conniot J., Silva J.M., Fernandes J.G., Silva L.C., Gaspar R., Brocchini S., Florindo H.F., Barata T.S. (2014). Cancer immunotherapy: Nanodelivery approaches for immune cell targeting and tracking. Front. Chem..

[65] Colzani B., Pandolfi L., Hoti A., Iovene P.A., Natalello A., Avvakumova S., Colombo M., Prosperi D. (2018). Investigation of antitumor activities of trastuzumab delivered by PLGA nanoparticles. Int. J. Nanomed..

[66] Studies G. (2014). PLGA-Based Nanoparticles in Cancer Immunotherapy and Immunomonitoring: A Versatile Vehicle for Targeting of Dendritic Cells.

[67] Cruz L.J., Tacken P.J., Fokkink R., Joosten B., Stuart M.C., Albericio F., Torensma R., Figdor C.G. (2010). Targeted PLGA nano- but not microparticles specifically deliver antigen to human dendritic cells via DC-SIGN in vitro. J. Control. Release.

[68] Bandyopadhyay A., Fine R.L., Demento S., Bockenstedt L.K., Fahmy T.M. (2011). The impact of nanoparticle ligand density on dendritic-cell targeted vaccines. Biomaterials.

[69] Rosalia R.A., Cruz L.J., van Duikeren S., Tromp A.T., Silva A.L., Jiskoot W., de Gruijl T., Lowik C., Oostendorp J., van der Burg S.H. (2015). CD40-targeted dendritic cell delivery of PLGA-nanoparticle vaccines induce potent anti-tumor responses. Biomaterials.

[70] Zhang Z., Tongchusak S., Mizukami Y., Kang Y.J., Ioji T., Touma M., Reinhold B., Keskin D.B., Reinherz E.L., Sasada T. (2011). Induction of anti-tumor cytotoxic T cell responses through PLGA-nanoparticle mediated antigen delivery. Biomaterials.

[71] Luo L., Zhu C., Yin H., Jiang M., Zhang J., Qin B., Luo Z., Yuan X., Yang J., Li W. (2018). Laser Immunotherapy in Combination with Perdurable PD-1 Blocking for Treatment of Metastatic Tumor. ACS Nano.

[72] Yuba E., Kanda Y., Yoshizaki Y., Teranishi R., Harada A., Sugiura K., Izawa T., Yamate Z., Sakaguchi N., Koiwai K. (2015). PH-sensitive polymer-liposome-based antigen delivery systems potentiated with interferon-γ gene lipoplex for efficient cancer immunotherapy. Biomaterials.

[73] Yoshizaki Y., Yuba E., Komatsu T., Udaka K., Harada A., Kono K. (2016). Improvement of peptide-based tumor immunotherapy using pH-sensitive fusogenic polymer-modified liposomes. Molecules.

[74] Yuba E., Uesugi S., Yoshizaki Y., Harada A., Kono K. (2017). Potentiation of cancer immunity-inducing effect by pH-sensitive polysaccharide-modified liposomes with combination of TGF-βtype I receptor inhibitor-embedded liposomes. Med. Res. Arch..

[75] Yuba E., Yamaguchi A., Yoshizaki Y., Harada A., Kono K. (2017). Bioactive polysaccharide-based pH-sensitive polymers for cytoplasmic delivery of antigen and activation of antigen-specific immunity. Biomaterials.

[76] Koshy S.T., Cheung A.S., Gu L., Graveline A.R., Mooney D.J. (2017). Liposomal Delivery Enhances Immune Activation by STING Agonists for Cancer Immunotherapy. Adv. Biosyst..

[77] Kranz L.M., Diken M., Haas H., Kreiter S., Loquai C., Reuter K.C., Meng M., Fritz D., Vascotto F., Hefesha H. (2016). Systemic RNA delivery to dendritic cells exploits antiviral defence for cancer immunotherapy. Nature.

[78] Sato Y., Hatakeyama H., Sakurai Y., Hyodo M., Akita H., Harashima H. (2012). A pH-sensitive cationic lipid facilitates the delivery of liposomal siRNA and gene silencing activity in vitro and in vivo. J. Control. Release.

[79] Hanafy N.A.N., El-Kemary M., Leporatti S. (2018). Micelles structure development as a strategy to improve smart cancer therapy. Cancers.

[80] Yuba E., Sakaguchi N., Kanda Y., Miyazaki M., Koiwai K. (2017). pH-Responsive Micelle-Based Cytoplasmic Delivery System for Induction of Cellular Immunity. Vaccines.

[81] Peng J., Xiao Y., Li W., Yang Q., Tan L., Jia Y., Qu Y., Qian Z. (2018). Photosensitizer Micelles Together with IDO Inhibitor Enhance Cancer Photothermal Therapy and Immunotherapy. Adv. Sci..

[82] Li H., Li Y., Wang X., Hou Y., Hong X., Gong T., Zhang Z., Sun X. (2017). Rational design of polymeric hybrid micelles to overcome lymphatic and intracellular delivery barriers in cancer immunotherapy. Theranostics.

[83] Liu L., He H., Liang R., Yi H., Meng X., Chen Z., Pan H., Ma Y., Cai L. (2018). ROS-Inducing Micelles Sensitize Tumor-Associated Macrophages to TLR3 Stimulation for Potent Immunotherapy. Biomacromolecules.

[84] Kong F.Y., Zhang J.W., Li R.F., Wang Z.X., Wang W.J., Wang W. (2017). Unique roles of gold nanoparticles in drug delivery, targeting and imaging applications. Molecules.

[85] Liu Y., Crawford B.M., Vo-dinh T. (2018). Gold nanoparticles-mediated photothermal therapy and immunotherapy. Immunotherapy.

[86] Almeida J.P.M., Figueroa E.R., Drezek R.A. (2014). Gold nanoparticle mediated cancer immunotherapy. Nanomed. Nanotechnol. Biol. Med..

[87] Evans E.R., Bugga P., Asthana V., Drezek R. (2018). Metallic nanoparticles for cancer immunotherapy. Mater. Today.

[88] Muddineti O.S., Ghosh B., Biswas S. (2015). Current trends in using polymer coated gold nanoparticles for cancer therapy. Int. J. Pharm..

[89] Kodiha M., Wang Y.M., Hutter E., Maysinger D., Stochaj U. (2015). Off to the organelles—Killing cancer cells with targeted gold nanoparticles. Theranostics.

[90] Dykman L.A., Staroverov S.A., Fomin A.S., Khanadeev V.A., Khlebtsov B.N., Bogatyrev V.A. (2018). Gold nanoparticles as an adjuvant: Influence of size, shape, and technique of combination with CpG on antibody production. Int. Immunopharmacol..

[91] Almeida J.P.M., Lin A.Y., Figueroa E.R., Foster A.E., Drezek R.A. (2015). In vivo gold nanoparticle delivery of peptide vaccine induces anti-tumor immune response in prophylactic and therapeutic tumor models. Small.

[92] Lin A.Y., Almeida J.P., Bear A., Liu N., Luo L., Foster A.E., Drezek R.A. (2013). Gold Nanoparticle Delivery of Modified CpG Stimulates Macrophages and Inhibits Tumor Growth for Enhanced Immunotherapy. PLoS ONE.

[93] Meir R., Shamalov K., Sadan T., Motiei M., Yaari G., Cohen C.J., Popovtzer R. (2017). Fast Image-Guided Stratification Using Anti-Programmed Death Ligand 1 Gold Nanoparticles for Cancer Immunotherapy. ACS Nano.

[94] Bear A.S., Kennedy L.C., Young J.K., Perna S.K., Mattos Almeida J.P., Lin A.Y., Eckels PC., Drezek R.A., Foster A.E. (2013). Elimination of Metastatic Melanoma Using Gold Nanoshell-Enabled Photothermal Therapy and Adoptive T Cell Transfer. PLoS ONE.

[95] Engle K.M., Mei T.-S., Wasa M., Yu J.-Q. (2008). NIH Public Access. Acc. Chem. Res..

[96] Visaria R.K. (2006). Enhancement of tumor thermal therapy using gold nanoparticle-assisted tumor necrosis factor- delivery. Mol. Cancer Ther..

[97] Zhao Y., Zhao X., Cheng Y., Guo X., Yuan W. (2018). Iron Oxide Nanoparticles-Based Vaccine Delivery for Cancer Treatment. Mol. Pharm..

[98] Zanganeh S., Hutter G., Spitler R., Lenkov O., Mahmoudi M., Shaw A., Pajarinen J.S., Nejadnik H., Goodman S., Moseley M. (2016). Iron oxide nanoparticles inhibit tumour growth by inducing pro-inflammatory macrophage polarization in tumour tissues. Nat. Nanotechnol..

[99] Hoang M.D., Lee H.J., Lee H.J., Jung S.H., Choi N.R., Vo M.C., Nguyen-pham T.N., Kim H.J., Park I.K., Lee J.J. (2015). Branched Polyethylenimine-Superparamagnetic Iron Oxide Nanoparticles (bPEI-SPIONs) Improve the Immunogenicity of Tumor Antigens and Enhance Th1 Polarization of Dendritic Cells. J. Immunol. Res..

[100] Kojima C., Kameyama R., Yamada M., Ichikawa M., Waku T., Handa A., Tanaka N. (2015). Ovalbumin Delivery by Guanidine-Terminated Dendrimers Bearing an Amyloid-Promoting Peptide via Nanoparticle Formulation. Bioconjug. Chem..

[101] Jain N.K., Tare M.S., Mishra V., Tripathi P.K. (2015). The development, characterization and in vivo anti-ovarian cancer activity of poly(propylene imine) (PPI)-antibody conjugates containing encapsulated paclitaxel. Nanomed. Nanotechnol. Biol. Med..

[102] Li K., Chang S., Wang Z., Zhao X., Chen D. (2015). A novel micro-emulsion and micelle assembling method to prepare DEC205 monoclonal antibody coupled cationic nanoliposomes for simulating exosomes to target dendritic cells. Int. J. Pharm..

[103] De La Peña H., Madrigal J.A., Rusakiewicz S., Bencsik M., Cave G.W.V., Selman A., Rees R.C., Travers P.J., Dodi I.A. (2009). Artificial exosomes as tools for basic and clinical immunology. J. Immunol. Methods.

[104] Chen W., Qin M., Chen X., Wang Q., Zhang Z., Sun X. (2018). Combining photothermal therapy and immunotherapy against melanoma by polydopamine-coated Al2O3nanoparticles. Theranostics.

[105] Song W., Kuang J., Li C.X., Zhang M., Zheng D., Zeng X., Lui C., Zhang X.Z. (2018). Enhanced Immunotherapy Based on Photodynamic Therapy for Both Primary and Lung Metastasis Tumor Eradication. ACS Nano.

[106] Hoopes P.J., Wagner R.J., Duval K., Kang K., Gladstone D.J., Moodie K.L., Crary-Burney M., Ariaspulido H., Veliz F.A., Steinmetz N.F. (2018). Treatment of Canine Oral Melanoma with Nanotechnology-Based Immunotherapy and Radiation. Mol. Pharm..

[107] Liu Q., Chen F., Hou L., Shen L., Zhang X., Wang D., Huang L. (2018). Nanocarrier-Mediated Chemo-Immuno Therapy Arrested Cancer Progression and Induced Tumor Dormancy in Desmoplastic Melanoma. ACS Nano.

